# The Potential of Combining Faba Bean (*Vicia faba* L.) and Pea Pod (*Pisum sativum* L.) Flours to Enhance the Nutritional Qualities of Food Products

**DOI:** 10.3390/foods14132167

**Published:** 2025-06-21

**Authors:** Khaoula Ben Said, Amel Hedhili, Sihem Bellagha, Hela Gliguem, Marie Dufrechou

**Affiliations:** 1 Higher School of Food Industries, University of Carthage, 58, Street of Alain Savary, Tunis 1003, Tunisia; 2 Valorization of Tunisian Natural Resources and Food Heritage Through Innovation, Research Unit PATIO, UR17AGR01, National Institute of Agronomy of Tunisia, University of Carthage, Tunis 1082, Tunisia; sihem.bellagha@inat.ucar.tn (S.B.); hela.gliguem@inat.ucar.tn (H.G.); 3 USC 1422 GRAPPE, L’Ecole Supérieure des Agricultures (ESA), INRAE, SFR QUASAV, 55 rue Rabelais, 49007 Angers, France; a.hedhili@groupe-esa.com

**Keywords:** pea pods, by-products, faba beans, thermal treatment, anti-nutritional factors, in vitro protein digestibility

## Abstract

Legumes have been identified as a key element of food innovation and excellent candidates for ensuring sustainability in food systems. However, certain legumes, such as faba beans and legume by-products, such as pea pods, are currently mainly being used in animal feed rather than exploited and valued in human nutrition. In this study, the nutritional properties, anti-nutritional factors, and in vitro protein digestibility of pea pod flour and raw and thermally treated (80, 120, 150, and 180 °C during 30 min) faba bean flours were investigated. For pea pod flours, the results showed a very interesting protein content (12.13%) and insoluble fibers (37.45%), as well as appreciable amounts of minerals, mainly calcium, potassium, magnesium, manganese, and iron. For faba bean flours, thermal treatment did not significantly affect the crude protein, ash, starch, and fat contents of the processed beans. Meanwhile, compared with raw faba bean flours, thermal treatment significantly decreased insoluble dietary fibers, anti-nutritional factors such as phytic acid, tannins, trypsin inhibitors, and alpha-galactosides and progressively improved the in vitro protein digestibility by 7,7%. In conclusion, faba bean and pea pod flours show significant potential as novel ingredients in the food industry. Their combination will enable the development of protein, fiber, and mineral-rich food products.

## 1. Introduction

Animal-based diets are undeniably protein-rich diets but are also directly linked to threats to global sustainability in food provision for present and future generations. Meat and dairy industries have been proven as determining factors in worsening the effects of climate change, including biodiversity loss, ecosystem imbalance, water depletion, and also greenhouse gas emissions (GHG). Moreover, human health concerns are commonly reported to be one of the key reasons for an urgent need to shift food production systems and dietary habits [1]. Consequently, calls for the integration of plant-based protein diets have emerged, aligning with greater demand in the plant protein market and a growing consciousness and awareness among consumers about the food they consume. The goal of many organizations is to adopt strategies to reduce the ratio of animal protein-to-plant protein consumption from 60:40 to 40:60 by 2050 [2]. The implementation of this change has become a popular subject of scientific analysis and debate, where the majority acknowledges the benefits of transitioning to these diets [3,4]. In this regard, legumes grown worldwide have gained more attention and are considered to be a sustainable alternative to meet this challenge due to their high protein content, which ranges from 17 to 40% depending on the species [5]. They are also rich and inexpensive sources of dietary fibers, minerals, carbohydrates, vitamins, and bioactive molecules [6,7,8,9]. In addition to their nutritional value, several positive health effects have been attributed to a diet rich in legumes, such as reducing the risks of chronic and cardiovascular diseases, increasing satiety, and improving glycemic control [5,10,11,12]. In addition, the inclusion of legumes in farming systems can contribute to some aspects of sustainability, such as reducing greenhouse gas emissions and improving soil quality due to the symbiotic fixation of atmospheric nitrogen [13,14].

Faba bean (*Vicia faba* L.) is an important crop from nutritional, economic, and ecological perspectives [15]. It not only offers a versatile and highly nutritious source of inexpensive protein for healthy food innovation [16] but also contributes to reducing the need for synthetic fertilizers and improving soil quality [17]. Nevertheless, faba beans are still insufficiently used in human nutrition in some countries. Their unappreciated taste in some cases or the presence of anti-nutritional compounds such as lectins, digestive enzyme inhibitors, condensed tannins, alpha-galactosides, phytic acid, vicine, and convicine may be among the reasons for this [9,15,18]. These anti-nutritional compounds can lead to health concerns such as decreased protein digestibility, a deficiency in minerals, or certain forms of digestive discomfort like flatulence and favism in some cases [19,20,21]. The effect of these anti-nutritional compounds on consumer’s health and preferences may be overcome by applying various processes such as germination, extrusion, roasting, infrared heating, dehulling, microwave heating, and fermentation. [22,23,24]. Among these processes, thermal processing has been shown to be an effective method for improving the quality of faba bean proteins by reducing their anti-nutritional compounds and decreasing the undesirable off-flavors generated by endogenous enzymes responsible for the unappreciated faba bean taste [25,26].

In addition to faba beans, this study also focuses on pea pods, a legume by-product generated from pea processing that represents about 40% of waste [27]. This by-product is generally blanched and then dried to obtain flour [27,28,29].

Pea pods have a favorable nutritional composition for human consumption due to their particular characteristics. They are rich in dietary fibers (59%; [30]), protein (14%; [29]), vitamins such as B1 and C (1.61 mg and 34.65 mg per 100 g DM, respectively [31]), minerals like iron and zinc (10 mg and 1.6 mg per mg DM, respectively [32]), and carotenoids (7.63 mg/100 g DM; [27]). Additionally, they are low-fat, low-calorie, and cholesterol-free [33]. Pea pods have also been reported to have positive health-promoting properties and numerous pharmacological benefits, such as strong antidiabetic activity and protective effects against alloxan-induced hepatic, renal, and reproductive damage, improving cardiovascular health, and cancer prevention [31,34]. Despite their abundance, pea pods have been largely overlooked for their potential use in human nutrition [30]. Their incorporation as food ingredients remains largely underexplored, as they are currently used mainly for animal feed. However, there has been recent interest in utilizing pea pods as valuable substrates for bioethanol production [35] and for the recovery or conversion of valuable compounds such as polysaccharides and carbohydrates [36,37]. The potential of these by-products in food systems remains largely unexplored, representing a missed opportunity to utilize this nutrient-rich resource for sustainable human consumption. As previous studies have addressed each of these two biomasses independently, to date, no work has explored their combined nutritional and functional potential to improve food formulations. In line with the concept of “crossing two hurdles with one leap”, the present study investigates these two highly nutritious, sustainable, and underutilized legume-based biomasses, faba beans and pea pods, within a unified framework. This integrated approach will provide new insights into the future dual utilization of faba bean and pea pod flours, strengthening their potential incorporation into sustainable and health-oriented food systems. Additionally, it will serve as a foundational first step toward developing an innovative, affordable, high-protein, high-fiber, low-fat, and gluten-free food product.

To achieve this, the objectives of the present study are twofold: first, we aim to investigate the functional and nutritional qualities of these two underutilized and sustainable ingredients, pea pod flour and non-thermally and thermally treated faba bean flours, and second, we aim to identify the complementary potential of these two ingredients with respect to their functional and nutritional qualities for better utilization.

## 2. Materials and Methods

### 2.1. Plant Materials and Sample Preparation

Green mature peas (*Pisum sativum* L.) and faba beans (*Vicia faba*, var minor) were purchased from a Tunisian local producer during the March-April (2022) period. The pea pods were obtained by manually removing green peas. The pea pods were washed several times with water to remove dust and extraneous matter, blanched for 2 min at 100 °C, and cooled down with tap water for 4 min to decrease temperature. Pea pods were dried in a ventilated oven (Memmert, France) at 45 °C for 6 h, ground in a mixer grinder for 2 min, and then sieved through a 500 µm mesh screen [28].

For the faba beans, thermal treatment was assessed at different temperatures. Four samples of whole faba bean seeds (250 g) were dry-heated in a hot air oven at 80 °C, 120 °C, 150 °C, and 180 °C for 30 min for each sample and then cooled down at room temperature. The raw seeds (non-thermally treated seeds) and four thermally treated seeds were ground to flour with a mixer grinder (Thermomix, VorwerkVkm, France) and then sieved with a 500 µm mesh screen. All samples were then stored at 4 °C until analyses.

### 2.2. Proximate Composition

The dry matter of the pea pods and the faba bean flours were determined according to the AOAC method (AOAC, 1997). Ash content was determined by incineration at 500 °C in a muffle furnace (Nabertherm, France) (AOAC, 1998). The total nitrogen content was analyzed by the Kjeldahl method with a conversion factor of 6.25 to calculate the total protein content (AOAC, 1995). Total insoluble fibers (hemicellulose, cellulose, and lignin) were determined according to the method of Soest [38] using the Fibersac system (Ankom Technology, Couëron, France). The total starch content of flour was determined using a commercial kit (K-TSHK, Megazyme, Bray, Ireland) and was calculated in g/100 g of dry matter according to the manufacturer’s instructions. Fat was extracted with a petroleum ether solvent with a Soxtec^TM^8000 apparatus (FOSS Analytical, Nanterre, France). All analyses were performed in triplicates.

### 2.3. Mineral Analysis

The mineral composition of pea pods and faba bean flours for major and trace elements was performed by X-ray fluorescence spectrometry (XRF) using a Thermo Scientific device Niton FXL FM-XRF (Thermo Fisher Scientific, Boston, MA, USA). spectrometer with a 50 kV, 200 μA X-ray tube and CDD GOLDD drift detector technology. The analysis was performed at the Mineralogical Laboratory, Faculty of Sciences, Sfax University. Analyses were performed in triplicates.

### 2.4. Phytochemical Composition

#### 2.4.1. Extraction Procedure

The method reported by Mejri et al. [32] was used with slight modifications. In total, 1 g of flour was mixed with 10 mL of methanol (80%). The mixture was sonicated for 5 min using an ultrasonic cleaning bath (Fisherbrand, FB15055, France), operating at a 42 kHz frequency and 80 W, and it was then left at room temperature in a dark place with stirring for 300 rpm during 24 h. The extracts were centrifuged at 4500 rpm for 10 min, and the supernatants were collected and stored at −20 °C until analyses. Absorbance measurements were made with a sequential analyzer (Gallery, ThermoFisher Scientific, Carnin, France).

#### 2.4.2. Determination of Total Phenolic Content

The total phenolic content (TPC) was carried out using the colorimetric Folin–Ciocalteu method [39]. In total, 25 µL of each extract was mixed with 125 µL of a 10-fold-diluted Folin–Ciocalteu reagent; then 100 µL of 7.5% Na_2_CO_3_ was added. After 30 min of incubation, the absorbance was measured at 750 nm, and total phenolic content was expressed as milligrams of Gallic Acid Equivalents (GAEs) per 100 g of dry matter. Analyses were performed in triplicates.

#### 2.4.3. Determination of Antioxidant Activity

The antioxidant activities of flour samples were determined using two complementary assays, including ferric reducing antioxidant power (FRAP) and 2,2diphenyl-1-picrylhydrazyl (DPPH) radical scavenging.

The FRAP was determined using the method of Benzie and Strain [40]. The assay was evaluated as the capacity to reduce the ferric ion (Fe^3+^) to the ferrous ion (Fe^2+^). The FRAP reagent was prepared by mixing 300 mM of acetate buffer at pH 3.6, 10 mM of 2,4,6-Tripyridyl-S-Triazine (TPTZ) in 40 mM HCl, and 20 mM of FeCl_3_ solution at a ratio of 10:1:1. In total, 150 µL of the sample extract was mixed with FRAP solution until a final volume of 3 mL was obtained and incubated for 30 min at 37 °C. The absorbance was measured at 593 nm with a UV spectrophotometer (SAFAS Monaco, France).

The DPPH radical-scavenging activity of the extracts was carried out by Yen et al. [41]. In total, 0.1 mL of the sample extract was prepared at different concentrations and added to 3.9 mL (0.025 g/L) of DPPH solution. The solution was incubated at room temperature for 60 min, and the decrease in absorbance at 515 nm was determined at the end of the incubation period. DPPH radical scavenging activity was expressed as the % calculated using Equation (1).

(1) %*radical scavenging activity* = 100 ∗ (*A*0 − *A*)/*A*0

where *A*0 is the absorbance of the control, and *A* is the absorbance of the sample extract.

For the DPPH and FRAP antioxidant activities, Trolox (T) was used as a standard, and the results were expressed as the mg of Trolox equivalent (TE) per 100 g of dry matter. All analyses were performed in triplicates.

### 2.5. Analyses of Anti-Nutritional Factors

#### 2.5.1. Determination of Condensed Tannins

The estimation of condensed tannins was performed using the vanillin assay with minor modifications [42]. One gram of each flour sample was added to 20 mL of a 1% HCl solution in methanol, vortexed, and incubated for 20 min at 30 °C in a water bath. Samples were then centrifuged at 4500 rpm for 10 min. The condensed tannins content was estimated by adding 1 mL of the sample extract to 5 mL of a vanillin reagent (0.5%, (*w*/*v*), vanillin in a 2% HCl in methanol) before being vortexed and incubated for 20 min at 30 °C. The absorbance was measured at 500 nm with a UV spectrophotometer (SAFAS Monaco, France). A 4% HCl solution in methanol was used as a blank, and catechin was used to prepare a linear standard curve. The results were presented in terms of mg catechin equivalents per 100 g of dry matter. Analyses were performed in triplicates.

#### 2.5.2. Determination of Trypsin Inhibitors

The trypsin inhibitor content was determined according to the method described by Kakade et al. [43]. Samples were extracted with 50 mL NaOH (0.01 N) for 1 h under agitation at room temperature; they were centrifuged at 4500 rpm for 10 min, and then the supernatants were collected and diluted by a factor of 1:10 using deionized water. In total, 2 mL of each diluted sample extract was placed in a tube test, and 2 mL of trypsin solution (4 mg in 200 mL of 0.001 HCl) was added. The mixture was then placed in a water bath at 37 °C for 10 min before adding 5 mL of BAPNA solution (80 mg in 2 mL of dimethyl sulfoxide diluted to 200 mL and pre-heated to 37 °C, 8.2 pH, tris-buffer). After 10 min of incubation, 1 mL of 30% acetic acid was added to stop the reaction. A blank was prepared following the same procedure, except that the trypsin solution was added after stopping the reaction by adding acetic acid. The absorbance values of the samples were measured at 410 nm with a UV spectrophotometer (SAFAS Monaco, France), and trypsin inhibitory activity (TIA) was defined in terms of the trypsin units inhibited (TUI) per gram of dry matter. Analyses were performed in triplicates.

#### 2.5.3. Phytic Acid Content

The analysis of the phytic acid content of the flour samples was performed using a Megazyme Phytate/Total Phosphorus kit(Megazyme, Vindry sur Turdine, France). After the sample extraction in 1 M HCl at room temperature overnight, the determination of phytic acid content was performed according to the manufacturer’s instructions. The results were presented in terms of mg of phytic acid per 100 g of dry matter. Analyses were performed in triplicates.

#### 2.5.4. Galactosyl-Sucrose Oligosaccharides Content

The alpha-galactosides content was evaluated following the instructions supplied by the raffinose/sucrose/D-glucose kit from Megazyme by measuring the galactosyl-sucrose-oligosaccharides (GSOs).

GSOs were first hydrolyzed to D-galactose, D-glucose, and D-fructose using α-galactosidase and invertase and then determined using a glucose oxidase/peroxidase reagent. α-GOSs were expressed as the grams of GSO per 100 g of dry matter. Analyses were performed in triplicate.

### 2.6. In Vitro Protein Digestibility (IVPD)

Pea pod and faba bean (raw and thermally treated) flours were subjected to an in vitro digestion protocol according to the standardized international consensus protocol INFOGEST reported by Brodkorb et al. [44] with some modifications. Firstly, for the oral phase, the amount of each sample corresponding to 0.5 g of protein was mixed with 1.6 mL of simulated salivary fluid (SSF), 10 µL of 0.3 M CaCl_2_, and 190 µL of Milli-Q water. Then, 200 µL of a freshly prepared enzyme mixture of human salivary α-amylase solution (75 U/mL) was added, and the mixture was incubated for 2 min at 37 °C under continuous stirring at 150 rpm. Then, to start the gastric phase, the endpoint of the oral phase was mixed with 3.2 mL of simulated gastric fluid (SGF) and 10 µL of 0.3 M CaCl_2_. The pH was adjusted to 3.0 using a 4 M HCl solution before the addition of 200 µL of a pepsin solution (2000 U/mL). The gastric digesta was incubated at 37 °C with continuous shaking (150 rpm) for 2 h. The intestinal phase was elaborated by mixing the gastric chime with 3.4 mL of simulated intestinal fluid (SIF) and 16 µL of 0.3 M CaCl_2._ The pH was adjusted to 7.0 with 1 M NaOH and 2 mL of pancreatin solution (100 U/mL); 1 mL of bile extract was also added to the mixture. Following 2 h of incubation at 37 °C under constant stirring, 50 µL of Pefabloc solution (0.1 M) was added to stop the overall digestion stages. The digests were centrifuged at 8000× *g* for 12 min at 4 °C, and supernatants that contained the soluble potential proteins fraction were collected and stored at −20 °C until analysis. A blank control was carried out by replacing the sample with 2 mL of Q-demineralized water. The protein content of samples before and after digestion was determined by the Kjeldahl method, and the in vitro protein digestibility (IVPD) was calculated according to Equation (2). Analyses were performed in triplicates.

(2) *IVPD*(%) = (*P*0 − *P*1)/*P*0 ∗ 100

where *P*0 is the initial protein content of the undigested sample; *P*1 is the protein content of the sample after digestion.

### 2.7. Determination of Functional Properties

#### Water and Oil Absorption Capacity

The water absorption capacity (WAC) of the flour was determined according to the method described by Noor Aziah and Komathi [45]. Briefly, 1 g of the flour sample was weighed in a centrifuged tube, and 10 mL of distilled water was added and mixed for 30 s. Samples were allowed to hydrate at room temperature for 2 h. The suspensions were then centrifuged (2800 rpm, 10 min), supernatants were discarded, and the weight of the hydrated sample was recorded. WAC was expressed as the g of water held per g of the dried sample. For the oil absorption capacity (OAC), the same protocol as mentioned above was followed, except that corn oil was used in place of distilled water. OAC was expressed as the g of oil held per g of the dried sample. Measurements were performed in triplicate.

### 2.8. Statistical Analysis

The data were analyzed by SPSS Statistics 20, using one-way analysis of variance (ANOVA) and Tukey’s test for multiple comparisons (significance level: *p*-value < 0.05). All values were expressed as the means ± standard deviation (SD).

## 3. Results

### 3.1. Nutritional Composition

The proximate composition of pea pod flour and both raw and thermally treated faba bean flour is presented in Table 1. The dry matter content of faba bean flour increased progressively during thermal treatment, ranging from 92.40 g/100 g in the raw sample to 99.37 g/100 g at 180 °C for 30 min. Statistically, significant differences (*p* < 0.05) were observed between the raw sample and those thermally treated, indicating moisture reduction with increasing temperature. Faba bean flour exhibited a consistently high protein content, averaging 32.22 ± 0.36 g/100 g DW, with no significant variations for the different thermal treatments (range 31.89 to 32 g/100 g DW) (Table 1). This indicates that the thermal process applied maintained the protein content, even at higher temperatures.

The ash content showed no significant impact of thermal treatment. Conversely, insoluble dietary fiber (IDF) showed a clear reduction with respect to temperature. In the raw sample, hemicellulose, cellulose, and lignin were measured at 23.08, 9.65, and 0.64 g/100 g DW, respectively. Thermal treatment led to a significant decrease in IDF, with the maximum loss (approximately 30%) occurring at 180 °C. Starch, a major carbohydrate constituent, ranged from 45.45 g/100 g DW in raw faba bean flour to 43.98 g/100 g in thermally treated faba bean flour at 180 °C, with no statistically significant differences among the samples. Similarly, fat content remained relatively unchanged under thermal treatment (1.57–1.63 g/100 g DW).

Pea pod flour also demonstrated valuable nutritional value (Table 1). It contained 12.13 g/100 g DW of protein and 3,98% of total ash. Notably, it had significant insoluble fiber content, comprising 16.86 g/100 g DW hemicellulose, 18.08 g/100 g DW cellulose, and 2.51 g/100 g DW lignin. In contrast to faba beans, their starch (8.76 g/100 g DW) and fat (1.48 g/100 g DW) contents were relatively low.

### 3.2. Phytochemical Composition

#### 3.2.1. Total Phenolic Content (TPC) and Antioxidant Activity

The TPC and antioxidant activities measured using DPPH and FRAP assays are reported in Table 2. For the faba bean flours, thermal treatment applied at temperatures above 120 °C resulted in a significant reduction in TPC from 152.79 to 139 mg GAE/100 g DW. Moreover, antioxidant activity decreased markedly following thermal treatment, with values decreasing from 156.64 to 117.86 mg ET/100 g DW (a 25% reduction) for DPPH and from 554.27 to 399.41 mgET/100 g DW (a decrease of 28%) for FRAP compared to raw faba bean flour. These observations underscore the sensitivity of antioxidant compounds to high-temperature processing.

Pea pod flour exhibited a promising TPC of 377.36 mg GAE/100 g DW. Its antioxidant activity was also prominent, with values of 267.96 mgET/100 g DW (DPPH) and 285.03 mgET/100 g DW (FRAP), underscoring its potential as a valuable ingredient.

#### 3.2.2. Mineral Composition

As shown in Table 3, potassium was the predominant macro-element in faba bean flours ranging from 1.60 to 1.64 g/100 g DW, followed by phosphorus (0.56–0.58 g/100 g DW) and magnesium (0.16–0.18 g/100 g DW). Among the trace elements, manganese (5.86–5.97 mg/100 g DW), zinc (4.9–4.96 mg/100 g DW), and iron (5–5.16 mg/100 g DW) were the most abundant. The thermal treatment process did not cause significant changes in the contents of either macro or micro-elements, highlighting the mineral stability of faba bean flours when exposed to thermal treatment.

Pea pod flour also demonstrated a favorable mineral profile. Calcium, potassium, and magnesium were identified as the main prominent macro-elements, while manganese (4.66 mg/100 g DW), iron (2.46 mg/100 g DW), and zinc (0.55 mg/100 g DW) were the dominant trace elements (Table 3). These results emphasize the nutritional richness of both matrices.

### 3.3. Anti-Nutritional Factors (ANFs)

The composition of anti-nutritional factors (ANFs) in pea pod flour, as well as the effect of thermal treatment on faba bean flours, are depicted in Table 4.

Thermal treatment led to a significant reduction in all measured anti-nutritional factors, highlighting its efficacy in enhancing the nutritional quality of faba bean flours. Condensed tannins decreased significantly by about 50%, from 362.54 mg/100 g DW in the raw sample to 184.18 mg/100 g DW in the thermally treated sample at 180 °C. The phytic acid content decreased from 1.25 to 0.90 g/100 g DW at 180 °C, while trypsin inhibitor activity decreased by nearly 52% from 0.92 to 0.44 TUI/mg DW. Additionally, a reduction of 44% was observed in alpha-galactosides, further confirming the effectiveness of thermal treatment in minimizing undesirable components.

The pea pod flour contained 226.90 mg/100 g DW of condensed tannins, 0.14 g/100 DW of phytic acid, 0.93 TIU/mg DW of trypsin inhibitors, and 0.73 g/100 g of α-galactosides DW.

### 3.4. In Vitro Protein Digestibility (IVPD)

The in vitro protein digestibility (IVPD) of the tested samples is given in Table 5. For faba bean flours, thermal treatment significantly enhanced the in vitro protein digestibility (IVPD), confirming its effectiveness in improving protein bioavailability. The raw faba bean flour exhibited an initial IVPD of 64.54%, which increased by 7,7% following thermal treatment at 180 °C. However, no significant differences were observed among the different thermally treated samples. In contrast, the in vitro protein digestibility (IVPD) of pea pod flour was in the order of 45.29%, reflecting its high fiber content.

### 3.5. Functional Properties

The functional properties of the different samples are presented in Table 6. For faba bean flour, thermal treatment has a significant impact on the water absorption capacity (WAC), with values increasing from 1.77 g/g DW in the raw sample to 2.39 g/g DW at 180 °C. In contrast, oil absorption capacity (OAC) remained mostly stable despite thermal treatment, maintaining a range between 1.55 and 1.69 g/g DW across all samples.

Pea pod flour demonstrated superior functional properties, with a WAC of 3.62 g/g and an OAC of 2.58 g/g.

## 4. Discussion

A key aspect of this study is to demonstrate the potential of the combined use of faba bean and pea pod flours, highlighting their complementary nutritional profiles in order to develop sustainable, functional food products.

One of the findings of this study is that thermal treatment did not significantly affect the protein content of faba bean flours. This underscores the viability of thermal treatment as a gentle processing strategy to preserve the nutritional value of faba bean protein. Furthermore, it confirms the observations of Guéguen et al. [46], who reported that dry heat treatments have a minimal impact on the native protein structure. More notably, this outcome emphasizes the higher protein concentration of faba bean flour compared to previous reports on various cultivars [6,17] and other commonly consumed legumes [47]. These results show the potential of faba bean flour as a valuable and concentrated plant-based protein source for nutritional applications.

As an underutilized legume by-product, pea pod flour has been scarcely studied for human food applications. In comparison to other by-products, such as soy husk (9 g/100 g DW; [48]) and cacao pod husk (8.6 g/100 g DW [49]), pea pod flour has a promising protein content, positioning this natural resource as a competitive candidate among agricultural residues. Its incorporation into human food, along with faba bean flour, could enhance nutritional profiles and promote sustainable valorization strategies.

Another important finding in this study is that ash content remained stable following thermal treatment. 

The effect of heat processes, such as extrusion or thermal treatment, on protein and ash contents remains inconsistent and inconclusive, varying with legume type and processing conditions. In their study, Korus et al. [50] reported both slight increases and decreases in the protein content in different bean varieties, while ash content generally remained unchanged or was slightly reduced. In the present work, the ash content of pea pod flour was within the range of previously reported amounts in the literature [32] but remained lower than in other studies [30,51]. These substantial differences may be attributed to varietal variations and differing environmental or agronomic conditions, including soil composition and climate.

Fiber composition is another key nutritional aspect that may be influenced by processing. In this regard, even after thermal treatment, faba bean flour samples retain appreciable amounts of IDF, confirming their relevance as a fiber-rich ingredient suitable for formulating high-fiber foods. As reported by Garcia-Amezquita et al. [52], processing factors, such as temperature and duration, directly affect the content of dietary fibers (both insoluble and soluble). Nevertheless, the observed reduction in IDF in this study may be partially explained by the thermal-induced solubilization of structural polysaccharides such as cellulose and hemicellulose into simpler carbohydrates, the hydrolysis of low-molecular-weight fiber fractions, or physical changes within the flour matrix. For pea pod flour, the recorded values revealed moderately high amounts of hemicellulose, cellulose, and lignin, which is consistent with its classification as a fiber-rich by-product. Although slightly lower than the values reported by Wadhwa et al. [35], the fiber content may have been reduced by processing steps such as the parchment removal and sieving procedure applied in the present study [28]. These steps likely yielded smoother and less fibrous pea pod flour, enhancing its sensory appeal and potential suitability for human consumption. Such a result aligns with increasing interest in transforming agricultural by-products into valuable food ingredients.

Despite the well-documented advantages of dietary fiber in terms of health benefits [6,31], most food products do not contain enough levels of insoluble dietary fiber in their composition [53]. Increasing fiber consumption is still a major public health goal, essential for disease prevention and overall wellbeing. In this context, the combined use of fiber-rich legumes and legume by-products as ingredients, such as pea pods and faba beans, presents a promising strategy to improve the nutritional quality of food products. This approach not only aligns with current dietary recommendations but also helps address key public health objectives related to insufficient fiber intake.

Another important finding in this study is the stability of starch content in faba bean flour after thermal treatment. This result is contrary to previous reports [54,55,56], which suggest that high temperatures often lead to the disruption of starch structures and, consequently, cause significant changes in their content amount. Typically, starch hydrolysis begins with a dry matter of approximately 35%, requiring a notable amount of water [57]. In the present study, however, the high dry matter content of both raw and thermally treated faba bean flours likely limited water availability, thereby restricting significant starch degradation. Additionally, the observed resistance to starch hydrolysis could be attributed to the intrinsic structural properties of pulse starches, which are known for their limited susceptibility to enzymatic degradation, as previously reported by Li et al. [58]. As for pea pod flour, considerably higher starch content was found compared to that previously reported [30], mainly due to varietal and environmental factors, which are known to significantly influence starch accumulation in plant tissues [59]. Beyond its various and important health benefits [60], starch also plays a determinant functional role in food systems. Its impact on textural and sensory qualities [61,62] is well documented, especially through thickening, gelatinization, and swelling properties.

Other macronutrients, such as fat, also play a role in defining the overall nutritional profile of legumes and their by-products. From a nutritional point of view, legumes are generally characterized by their low-fat content [63], and this was confirmed in the present study for both raw and thermized faba bean flours. The lack of significant changes after thermal treatment may be attributed to the formation of lipid–protein or lipid–starch (mainly amylose) complexes, which are known to resist conventional lipid extraction methods [64]. Regarding pea pod flour, the measured fat content varied compared to other studies [28,30], which may be explained by differences in cultivar, growing conditions, and agronomic practices. Notably, the naturally low-fat content of both faba bean and pea pod flour offers a significant advantage for product stability. High-fat amounts can lead to oxidation, often catalyzed by endogenous enzymes such as peroxidase and lipoxygenase, which produce volatile compounds responsible for off-flavors and a short food shelf life. Consequently, the low lipid amounts in these flours make them highly suitable for use in shelf-stable food formulations.

In addition to their favorable nutritional qualities, legumes stand out as a remarkable source of polyphenols, contributing significantly to dietary antioxidant intake. This study reveals that thermal treatment up to 120 °C did not significantly affect the total phenolic content of faba bean flours, supporting the thermal stability of phenolics under controlled heat treatment [65]. However, higher thermal treatment temperatures (150 and 180 °C) led to a marked decline in TPC, indicating the degradation of thermo-labile polyphenols or the polymerization of certain compounds, such as tannins, reducing their extractability and solubility [66]. This finding provides a practical insight into food processing, underscoring the need for carefully modulated processing to preserve bioactive compounds. This reduction in extractable phenolics at higher temperatures is reflected in the corresponding antioxidant activity measurements. As expected, a decrease in antioxidant activity, measured by DPPH and FRAP assays, aligns with the observed phenolic loss, highlighting their positive correlation with phenolic content [67]. These findings are in line with data reported by Siah et al. [68], who observed an approximately 50% reduction in DPPH activity in roasted faba beans at 150 °C.

Pea pod flour, on the other hand, showed a high amount of TPC, underscoring its value as a viable and promising source of natural bioactive content that can serve as an effective alternative to synthetic antioxidants in food systems, thereby supporting the development of clean-label products [69,70].

Together, these results emphasize the potential of combining faba bean and pea pod flours to improve the intake of health-promoting compounds in the diet while also contributing to the development of sustainable and functional food products.

Besides macronutrients and bioactive compounds, legumes are also a promising source of essential minerals.

Another key finding of this study is that the thermal treatment process did not appear to significantly affect the mineral profile of faba bean flours. Essential macro- and micro-elements such as potassium, phosphorus, manganese, iron, and zinc remain stable across heat treatments. However, it is important to note that the current scientific literature offers limited information regarding the sensitivity of legume minerals to thermal treatments. Further investigation is needed to clarify these interactions. Preserving mineral content during processing is critical for maintaining the nutritional value of legume-based ingredients, particularly for populations vulnerable to micronutrient deficiencies. Similarly, pea pod flour also demonstrated a rich mineral profile. Significant amounts of calcium, potassium, magnesium, and phosphorus were detected, along with notable concentrations of trace elements such as manganese, zinc, and iron. Such a composition reinforces the value of pea pod flour, not only as a sustainable ingredient derived from agro-industrial waste but also as a potential source of essential minerals.

Overall, the mineral composition of these two flours, which is even better if they are combined, supports their integration into food products and contributes to enhancing nutritional quality while addressing the fact that mineral deficiency may be prevalent in any population. Although the nutritional richness of legumes in macronutrients, bioactive compounds, and essential minerals has been determined, it is also important to consider the presence of ANFs, which may limit their nutritional potential in functional food applications.

As shown in Table 4, thermal treatment at moderate-to-high temperatures reduced the concentrations of all investigated ANFs in faba bean flours, suggesting that heat treatment may be a promising strategy to mitigate their negative effects. This reinforces the potential of thermal processing as a practical strategy to improve the nutritional quality of legume-based ingredients. The observed reduction in the condensed tannin content aligns with earlier reports of Abd El-Hady and Habiba [71], Khattab and Arntfield [72], and Arise et al. [73] for different legume species, attributing these reductions to the thermolabile nature of tannins, alterations in their chemical reactivity, or the formation of insoluble complexes during thermal treatment. As with condensed tannins, phytic acid, a major ANF found in legumes, impairs nutrient digestibility and bioavailability [15]. The reduced phytic acid amounts following thermal treatment aligns with previous findings across various legume species [71,74], which was mainly explained by the thermal degradation of inositol phosphate molecules to lower phosphate forms (tri-, tetra-, and pentaphosphates) [75].

In line with phytic acid and condensed tannins, thermal treatment proved to be an effective process to reduce trypsin inhibitor activity (TIA) even at relatively moderate thermal intensities. Such a result confirms the heat-sensitive nature of trypsin inhibitors, which has been consistently reported in the literature [23,65,73]. Nevertheless, the extent of TIA reduction largely depends on several process parameters, including temperature, the duration of heating, particle size, and moisture content [72,74,75,76].

To conclude the analysis of ANFs, α-GOS is the least studied group of compounds examined in this study. Compared to other legumes, such as peas, beans, and lentils [77], faba bean flour demonstrated a relatively moderate α-GOS content. Interestingly, thermization also proved effective in reducing α-GOS levels in faba bean flours, which is likely due to the heat hydrolysis of oligosaccharides into simpler disaccharides and monosaccharides, which is more digestible [72]. This result fills a gap in the current literature, where most reports focus on germination and soaking or enzymatic treatments for α-GOS reduction. Thus, this finding advances our understanding of heat pretreatments as a viable mitigation strategy for ANFs.

In parallel, the analysis of pea pod flour revealed relatively low amounts of ANFs, which reinforces its potential as a valuable ingredient in functional food development. Despite the growing nutritional interest in legumes and the increasing focus on upcycling agro-industrial by-products, there is limited comprehensive information on the presence and behavior of ANFs in pea pods. The profiling of condensed tannins, phytic acid, trypsin inhibitors, and α-GOS in this by-product brings new data to an underexplored area. Most existing studies have focused on peas, with little to no data on the specific anti-nutritional profile of these pods. Given the nutritional significance and potential health implications of these compounds, it is crucial to enhance our understanding of their occurrence in pea pod matrices. The identification and quantification of ANFs in pea pods are fundamental to determining their nutritional limitations.

Given that ANFs are known to impair nutrient absorption and interfere with protein utilization, their reduction may have further implications for improving protein quality. While protein content provides a measure of potential nutritional value, digestibility reflects the actual amount of protein that can be absorbed and utilized by the body [64]. Interestingly, the observed improvement in IVPD aligns with the results of the present study and those of previous ones [73,78,79], suggesting that it could be linked to the thermal reduction in anti-nutritional factors, namely condensed tannins, trypsin inhibitors, and phytic acid, which were well-documented for their ability to hinder protein digestion either by forming insoluble complexes with protein or by limiting enzyme accessibility through protein binding interactions.

Likewise, improved IVPD could be attributed to the decrease in IDF content [78]. Additionally, heat processing induces protein denaturation, which can lead to structural disintegration, thereby increasing the surface area, chain flexibility, and accessibility to enzymatic hydrolysis [80]. Furthermore, according to Kalpanadevi and Mohan [23], thermal treatment may also contribute indirectly to improving digestibility by promoting oligosaccharide solubility and diffusion, the activation of phytase enzymes, and the enhancement of endogenous α-galactosidase activity, all of which further alleviate digestion-inhibiting conditions.

Beyond thermal treatment, several studies [23,65,71,73,74, ,79,81,82,83,84] have investigated the impact of alternatives or complementary processing methods, such as germination, fermentation, soaking, and extrusion, on the digestibility of legume proteins. These treatments have shown considerable effectiveness in reducing both heat-stable and heat-sensitive ANFs and further enhancement in protein bioavailability. Therefore, the integration of thermal treatment with other processing methods may offer a promising strategy to optimize the nutritional profile of legume-based ingredients.

Although the increase in in vitro protein digestibility following thermal treatment was moderate (7.7%), it was statistically significant and might be biologically meaningful, particularly for populations that rely heavily on plant-based protein sources. Importantly, while in vitro methods offer valuable insights, future studies should focus on assessing amino acid bioavailability and conducting in vivo trials to better determine the nutritional impact of this enhancement in real dietary contexts.

In contrast, pea pod flour exhibited a markedly low IVPD, mainly due to its high fiber content [78]. The high fiber fraction likely restricts enzyme access to protein substrates; thus, specific pretreatments may be required if pea pods are to be fully valorized as a protein source.

Currently, there is a significant gap in the literature regarding the IVPD of pea pods, as most studies have primarily focused on the seeds and the impact of various processing treatments applied to them. However, it is important to note that pea pods still contain a considerable amount of protein, which could meaningfully contribute to overall nutritional intake if appropriately valorized. Therefore, further research is needed to assess their IVPD, explore methods to enhance them and evaluate their potential incorporation into food applications as a sustainable and nutritious ingredient.

To maximize the dietary benefits of legumes and their by-products, it is essential to gain a comprehensive understanding of both their nutritional and anti-nutritional properties. Additionally, evaluating the functional properties of legume flours such as WAC and OAC is important for their effective integration into food formulations. These functional properties influence the stability, texture, and sensory qualities of final food products. They are governed by the intricate interplay between molecular conformation and the physicochemical behavior of food ingredients throughout various stages, including processing, storage, preparation, and consumption [85]. Consequently, conducting a combined evaluation of the nutritional, anti-nutritional, and functional characteristics of faba bean and pea pod flours can provide a more complete perspective on their dual potential both in health promotion and food innovation.

This study confirmed that the thermal treatment process enhances the WAC of faba bean flour, especially at high processing temperatures (180 °C). This improvement can be attributed to several factors, including proximate composition, particle size, and distribution, as well as heat-induced physical changes [22,86]. Furthermore, protein quality, their conformational structure, and the availability of polar amino acids, which exhibit a high affinity for water molecules, play key roles in determining WAC alongside carbohydrate composition [87]. High temperatures may also damage starch granules, disrupt their crystalline structure, and increase the solubility and leaching of amylose, all of which contribute to greater water absorption [88]. Therefore, to better understand the mechanisms behind the increased WAC in thermally treated faba bean flours, further research focusing on the structural conformations of proteins and starch is recommended.

Unlike WAC, the OAC of faba bean flour remained largely unaffected by thermization since protein composition, and more specifically, the hydrophobic part of proteins, are the determinants of OAC [5,85]. Thus, this result suggests that thermal treatment did not notably alter protein hydrophobicity.

In contrast, pea pod flour exhibited both high WAC and OAC, highlighting its distinctive functional profile, especially considering its status as an underutilized by-product. The high WAC may be due to the presence of hydrophilic compounds like fibers and polysaccharides, which have a strong capacity to bind with water [89]. The high OAC, on the other hand, suggests the potential presence of non-polar amino acids that are capable of interacting with the hydrocarbon chains of fats. These combined properties are particularly advantageous in the formulation of various food products, including doughs, biscuits, and custards, where ingredients are expected to absorb water and/or oil without excessive protein solubilization to enhance texture and preserve flavor and mouthfeel [90].

It should be noted that this study can be subjected to certain limitations, particularly the use of raw materials from a single geographical location and season. However, it provides region-specific data that contribute to Mediterranean and North African food system characterization, where these contexts are often under-represented in the literature. In this sense, the use of Tunisian-grown legumes supports broader objectives related to food sovereignty and waste recovery. Future studies should consider comparisons with different cultivars and varieties of faba beans and pea pods from different regions of the world in order to assess variability in composition and functionality and to generalize the findings. Another limitation lies in the emphasis placed on compositional and in vitro digestibility assessments. Future investigation on sensory acceptance is needed, which will enable complete characterization with a view to incorporating such flours into innovative formulations that are highly nutritious and acceptable to consumers.

## 5. Conclusions

Due to their complementary nutritional and functional profiles, faba bean and pea pod flours hold promise for the development of sustainable- and health-oriented food products. This study brings new insights by characterizing, for the first time, the anti-nutritional factors and in vitro protein digestibility of pea pod flour. It also confirmed the efficiency of thermal treatment in reducing ANFs and improving IVPD. The combined use of these two flours improves the formulation of high-protein and high-fiber products.

Their integration into food formulations like snacks and plant-based alternatives could also help upgrade underutilized legumes and agro-industrial by-products, supporting more plant-based food systems. The dual valorization of faba beans and pea pods aligns with the targets of sustainability and improving public health nutrition. Further studies are required to understand the behavior of these flours in food applications regarding their functional and sensory properties to ensure consumer food acceptance.

## Figures and Tables

**Table 1:** Proximate composition of faba bean and pea pod flours (g/100 g on a dry weight (DW) basis).

Parameters	Faba Bean Flours					Pea Pod Flour
	Thermally Treated (30 min)					
	Raw	80 °C	120 °C	150 °C	180 °C	
Dry matter	92.40 ± 0.27 ^a^	96.04 ± 0.40 ^b^	99.27 ± 0.11 ^c^	99.33 ± 0.43 ^c^	99.37 ± 0.01 ^c^	91.72 ± 0.16
Protein	32.22 ± 0.36 ^ab^	32.12 ± 0.16 ^ab^	31.89 ± 0.10 ^a^	32.62 ± 0.33 ^b^	32 ± 0.16 ^ab^	12.13 ± 0.05
Ash	3.67 ± 0.05 ^a^	3.29 ± 0.10 ^b^	3.34 ± 0.01 ^b^	3.27 ± 0.07 ^b^	3.38 ± 0.08 ^b^	3.98 ± 0.05
Hemicellulose	23.08 ± 0.12 ^a^	20.62 ± 0.89 ^b^	19.77 ± 0.42 ^b^	18.02 ± 0.01 ^c^	16.47 ± 0.09 ^d^	16.86 ± 0.18
Cellulose	9.65 ± 0.33 ^a^	8.86 ± 0.51 ^ab^	8.44 ± 0.27 ^b^	6.58 ± 0.03 ^c^	6.61 ± 0.04 ^c^	18.08 ± 0.07
Lignin	0.64 ± 0.01 ^a^	0.62 ± 0.00 ^a^	0.62 ± 0.02 ^a^	0.57 ± 0.01 ^b^	0.55 ± 0.02 ^b^	2.51 ± 0.03
Total IDF	33.37	30.10	28.82	25.16	23.63	37.45
Starch	45.45 ± 0.97 ^a^	44.41 ± 2.35 ^a^	42.36 ± 1.29 ^a^	43.37 ± 2.37 ^a^	43.98 ± 0.72 ^a^	8.76 ± 0.03
Fat	1.63 ± 0.07 ^a^	1.60 ± 0.01 ^a^	1.58 ± 0.02 ^a^	1.59 ± 0.08 ^a^	1.57 ± 0.17 ^a^	1.48 ± 0.05

IDF: insoluble dietary fibers. Data are shown as the means ± SD from triplicate analysis. Values in the same line with different superscript letters are significantly different (*p* < 0.05).

**Table 2:** Phytochemical composition of faba bean and pea pod flours.

Parameters	Faba Bean Flours					Pea Pod Flour
	Thermally Treated (30 min)					
	Raw	80 °C	120 °C	150 °C	180 °C	
TPC (mg GAE/100 g DW)	152.79 ± 1.47 ^a^	152.49 ± 4.37 ^a^	147.50 ± 3.16 ^ab^	134.93 ± 1.77 ^c^	139 ± 4.69 ^bc^	377.36 ± 11.67
DPPH (mgET/100 g DW)	156.63 ± 3.66 ^a^	157.15 ± 9.46 ^a^	150.18 ± 4.23 ^a^	150.60 ± 3.45 ^a^	117.86 ± 9.49 ^b^	267.96 ± 1.29
FRAP (mgET/100 g DW)	554.27 ± 2.02 ^a^	533.07 ± 2.37 ^b^	501.89 ± 1.54 ^c^	461.20 ± 2.25 ^d^	399.41 ± 8.09 ^e^	285.03 ± 1.40

TPC: total phenolic content; DPPH: 2,2diphenyl-1-picrylhydrazyl; FRAP: ferric reducing antioxidant power. Data are shown as means ± SD from triplicate analysis. Means followed by different superscripts in the same line are significantly different (*p* < 0.05).

**Table 3:** Mineral composition of faba beans and pea pods flours * (g/100 g DW), ** (mg/100 g DW).

	Faba Beans					Pea Pods
	Thermized (30 min)					
	Raw	at 80 °C	at 120 °C	at 150 °C	at 180 °C	
Potassium *	1.64 ± 0.01 ^a^	1.62 ± 0.00 ^ab^	1.60 ± 0.00 ^b^	1.60 ± 0.01 ^b^	1.61 ± 0.01 ^b^	0.77 ± 0.02
Sodium *	0.02 ± 0.00 ^a^	0.02 ± 0.00 ^a^	0.02 ± 0.00 ^a^	0.02 ± 0.00 ^a^	0.02 ± 0.00 ^a^	0.23 ± 0.00
Calcium *	0.07 ± 0.01 ^a^	0.08 ± 0.00 ^a^	0.07 ± 0.01 ^a^	0.07 ± 0.00 ^a^	0.07 ± 0.00 ^a^	1.48 ± 0.00
Magnesium *	0.18 ± 0.00 ^a^	0.18 ± 0.01 ^ab^	0.17 ± 0.01 ^ab^	0.16 ± 0.01 ^b^	0.16 ± 0.01 ^b^	0.59 ± 0.01
Phosphorus *	0.57 ± 0.00 ^a^	0.58 ± 0.01 ^ab^	0.57 ± 0.00 ^b^	0.56 ± 0.01 ^b^	0.58 ± 0.00 ^a^	0.38 ± 0.01
Manganese **	5.93 ± 0.01 ^a^	5.89 ± 0.03 ^a^	5.86 ± 0.04 ^a^	5.92 ± 0.09 ^a^	5.97 ± 0.08 ^a^	4.66 ± 0.03
Zinc **	4.91 ± 0.01 ^a^	4.96 ± 0.04 ^a^	4.90 ± 0.03 ^a^	4.95 ± 0.05 ^a^	4.91 ± 0.00 ^a^	0.55 ± 0.01
Iron **	5.16 ± 0.02 ^a^	5.12 ± 0.06 ^a^	5 ± 0.02 ^b^	5.08 ± 0.04 ^ab^	5.12 ± 0.01 ^a^	2.46 ± 0.01

Data are shown as the means ± SD from triplicate analysis. Different letters (superscripts) in the same line indicate significant differences between values (*p* < 0.05).

**Table 4:** Anti-nutritional factors of faba bean (FB) (raw and thermally treated for 30 min) and pea pod flours.

Sample	Condensed Tannins	Phytic Acid	Trypsin Inhibitor	α-Galactosides
	(mg EC/100 g DW)	(g/100 g DW)	(TUI/mg DW)	(g/100 g DW)
Raw FB	362.54 ± 2.81 ^a^	1.25 ± 0.03 ^a^	0.92 ± 0.03 ^a^	4.04 ± 0.07 ^a^
Thermally treated FB at 80 °C	274.41 ± 6.83 ^b^	1.21 ± 0.02 ^ab^	0.90 ± 0.01 ^a^	3.70 ± 0.06 ^b^
Thermally treated FB at 120 °C	196.22 ± 4.99 ^c^	1.17 ± 0.02 ^b^	0.46 ± 0.02 ^b^	3.15 ± 0.12 ^c^
Thermally treated FB at 150 °C	186.89 ± 5.27 ^cd^	1.07 ± 0.04 ^c^	0.44 ± 0.01 ^b^	3.38 ± 0.26 ^c^
Thermally treated FB at 180 °C	184.18 ± 5.91 ^d^	0.90 ± 0.01 ^d^	0.49 ± 0.01 ^c^	2.26 ± 0.03 ^d^
Pea Pod	226.90 ± 2.41	0.14 ± 0.01	0.93 ± 0.01	0.73 ± 0.03

Data are shown as the means ± SD from triplicate analysis. Values in the same column followed by similar superscript letters are not significantly different at *p* < 0.05.

**Table 5:** In vitro protein digestibility (IVPD) of faba bean (FB) (raw and thermally treated for 30 min) and pea pod flours.

Sample	% IVPD
Raw FB	64.54 ± 0.07 ^a^
Thermally treated FB at 80 °C	71.63 ± 1.74 ^b^
Thermally treated FB at 120 °C	71.60 ± 0.57 ^b^
Thermally treated FB at 150 °C	71.48 ± 1.58 ^b^
Thermally treated FB at 180 °C	72.27 ± 0.82 ^b^
Pea pod	45.29 ± 1.81

Data are shown as the means ± SD from triplicate analysis. Means in the same column with different letters are significantly (*p* < 0.05) different.

**Table 6:** Functional properties of faba bean and pea pod flour.

Parameters	Faba Bean Flours					Pea Pod Flour
	Thermally Treated (30 min)					
	Raw	80 °C	120 °C	150 °C	180 °C	
Water absorption capacity (g water/g DW)	1.77 ± 0.02 ^a^	1.81 ± 0.01 ^b^	1.86 ± 0.01 ^b^	1.95 ± 0.01 ^c^	2.39 ± 0.01 ^d^	3.62 ± 0.07
Oil absorption capacity (g oil/g DW)	1.69 ± 0.07 ^a^	1.65 ± 0.01 ^a^	1.67 ± 0.04 ^a^	1.60 ± 0.02 ^a^	1.55 ± 0.04 ^b^	2.58 ± 0.01

Data are shown as the means ± SD from triplicate analysis and expressed on a dry weight (DW) basis. Values in the same line followed by similar superscript letters were not significantly different at *p* < 0.05.

## Data Availability

Data will be made available upon request.

## Abbreviations

GHG	Greenhouse Gases
DPPH	2,2diphenyl-1-picrylhydrazyl
FRAP	Ferric Reducing Antioxidant Power
TPTZ	2,4,6-Tripyridyl-S-Triazine
α-GOS	Alpha Galactosyl-Sucrose-Oligosaccharides
IVPD	In Vitro Protein Digestibility
SSF	Simulated Salivary Fluid
SGF	Simulated Gastric Fluid
SIF	Simulated Intestinal Fluid
TE	Trolox Equivalent
GAE	Gallic Acid Equivalents
EC	Equivalents Catechin
WAC	Water Absorption Capacity
OAC	Oil Absorption Capacity
IDF	Insoluble Dietary Fibers
DW	Dry Weight
TPC	Total Phenolic Content
ANFs	Anti-Nutritional Factors
FB	Faba Bean
TIA	Trypsin Inhibitor Activity
RFOs	Raffinose Family Oligosaccharides
